# Meckel's Diverticulum Diagnosed by Balloon-Assisted Enteroscopy: A Multicenter Report from the Taiwan Association for the Study of Small Intestinal Diseases (TASSID)

**DOI:** 10.1155/2021/9574737

**Published:** 2021-11-18

**Authors:** Jen-Wei Chou, Chen-Shuan Chung, Tien-Yu Huang, Chia-Hung Tu, Chen-Wang Chang, Chung-Hsin Chang, Yen-Po Wang, Wen-Hung Hsu, Hsu-Heng Yen, Chia-Jung Kuo, Chiao-Hsiung Chuang, Ching-Pin Lin, Tzung-Jiun Tsai, Ming-Yao Su, Horng-Yuan Wang, Deng-Chyang Wu, Cheng-Tang Chiu

**Affiliations:** ^1^Center for Digestive Medicine, Department of Internal Medicine, China Medical University Hospital, Taichung, Taiwan; ^2^Taiwan Association for the Study of Small Intestinal Diseases (TASSID), Taoyuan, Taiwan; ^3^Division of Gastroenterology and Hepatology, Department of Internal Medicine, Far Eastern Memorial Hospital, New Taipei City, Taiwan; ^4^Division of Gastroenterology, Tri-Service General Hospital, National Defense Medical Center, Taipei, Taiwan; ^5^Division of Gastroenterology and Hepatology, Department of Internal Medicine, National Taiwan University Hospital, Taipei, Taiwan; ^6^Division of Gastroenterology and Hepatology, Mackay Memorial Hospital, Taipei, Taiwan; ^7^Division of Gastroenterology and Hepatology, Department of Internal Medicine, Taichung Veterans General Hospital, Taichung, Taiwan; ^8^Division of Gastroenterology and Hepatology, Department of Internal Medicine, Taipei Veterans General Hospital, Taipei, Taiwan; ^9^Division of Gastroenterology, Department of Internal Medicine, Kaohsiung Medical University Hospital, Kaohsiung Medical University, Kaohsiung, Taiwan; ^10^Division of Gastroenterology, Department of Internal Medicine, Changhua Christian Hospital, Changhua, County, Taiwan; ^11^Department of Gastroenterology and Hepatology, Chang Gung Memorial Hospital at Linkou, Chang Gung University, School of Medicine, Taoyuan, Taiwan; ^12^Division of Gastroenterology and Hepatology, Department of Internal Medicine, National Cheng-Kang University Hospital, Tainan, Taiwan; ^13^Division of Gastroenterology and Hepatology, Department of Internal Medicine, Chung Shan Medical University Hospital, Taichung, Taiwan; ^14^Division of Gastroenterology and Hepatology, Department of Internal Medicine, Kaohsiung Veterans General Hospital, Kaohsiung, Taiwan; ^15^Department of Internal Medicine, New Taipei Municipal Tucheng Hospital, New Taipei City, Taiwan

## Abstract

**Background and Aims:**

Patients with Meckel's diverticulum (MD) are difficult to preoperatively diagnose because of its endoscopic inaccessibility. Balloon-assisted enteroscopy (BAE) allows endoscopic access to the entire small intestine. The aim of the current study was to investigate patients with MD diagnosed by BAE in Taiwan.

**Methods:**

We conducted a retrospective, multicenter study of patients with MD who were diagnosed by BAE in Taiwan. The clinical characteristics, endoscopic features, histopathological findings, treatment methods, and outcomes were analyzed.

**Results:**

A total of 55 patients with MD were enrolled (46 males and 9 females). The mean age at diagnosis was 34.1 years. Overt gastrointestinal bleeding (87.3%) was the primary indication for BAE, followed by abdominal pain (9.1%), suspected small bowel tumor (1.8%), and Crohn's disease follow-up (1.8%). The mean distance between the ileocecal valve and MD was 71.6 cm (regarding diagnostic yields: BAE—100%, capsule endoscopy—40%, Meckel's scan—35.7%, computed tomography—14.6%, small bowel series—12.5%, and angiography—11.1%; regarding endoscopic features of MD: a large ostium—89.1%, a small ostium—7.3%, and a polypoid mass—3.6%). Surgical treatment was performed in 76.4% patients, and conservative treatment was performed in 23.6% patients. The mean length of MD in 42 patients who underwent surgical resection was 5.2 cm (in 43 patients of MD with available histopathology: heterotopic gastric tissue, 42.4%, heterotopic gastric and pancreatic tissues, 7%; heterotopic pancreatic tissue, 4.7%; heterotopic colonic tissue, 2.3%; and a neuroendocrine tumor, 2.3%).

**Conclusions:**

The current study showed BAE is a very useful modality for detecting MD compared with other conventional modalities.

## 1. Introduction

Meckel's diverticulum, which was originally described by the German anatomist Johann Friedrich Meckel (the Younger) in 1809, is the result of incomplete atrophy of the omphalomesenteric duct [1]. As determined by previous autopsy reports, Meckel's diverticulum occurs in about 2-4% of the general population [2, 3]. Most patients with Meckel's diverticulum are asymptomatic during their lifetime; however, 4-6% of patients develop complications, including gastrointestinal (GI) bleeding, intestinal obstruction, intussusception, diverticulitis, enteroliths, perforation, fistula, and tumors [4–6]. A study by Mackey and Dineen found that 16.9% of patients with Meckel's diverticulum developed obvious symptoms [7]. In the past, preoperative diagnosis of Meckel's diverticulum has been a challenge for endoscopists. The main reasons for this difficulty are its deep location and the anatomical tortuosity of the small intestine, meaning there is poor endoscopic accessibility. Since the newly developed modality of balloon-assisted enteroscopy (BAE), including double-balloon enteroscopy (DBE) and single-balloon enteroscopy (SBE), was introduced, there have been some case reports in the English literature of patients with Meckel's diverticulum which has been diagnosed by BAE [8–10]. In Taiwan, DBE and SBE were first introduced in 2003 and 2007, respectively. The aim of the present study was to investigate the clinical characteristics, endoscopic features, histopathological findings, treatment methods, and clinical outcomes of patients with Meckel's diverticulum diagnosed by BAE in Taiwan.

## 2. Patients and Methods

We conducted a retrospective, multicenter study of patients with Meckel's diverticulum who were diagnosed by BAE in Taiwan between August 2005 and December 2020. Eligible patients were assessed at the following medical centers and hospitals which are affiliated members of the Taiwan Association of Small Intestinal Disorders (TASSID): China Medical University Hospital, Far Eastern Memorial Hospital, Chung Shan Medical University Hospital, Tri-Service General Hospital, National Taiwan University Hospital, National Cheng Kang University Hospital, Taipei Veterans General Hospital, Chang Gung Memorial Hospital at Linkou, Kaohsiung Medical University Hospital, Tainan Municipal Hospital, Kaohsiung Veterans General Hospital, MacKay Memorial Hospital, and Changhua Christian Hospital.

Antegrade or retrograde approach BAE, including DBE (Fujinon, Saitama, Japan) and SBE (Olympus, Tokyo, Japan), was performed on patients who had suspected small intestinal disorders at several hospitals in Taiwan. A total of 55 consecutive patients with Meckel's diverticulum as diagnosed by BAE were included in the current study. The clinical characteristics, endoscopic features, histopathological findings, treatment methods, and clinical outcomes of these patients were analyzed and discussed.

## 3. Statistical Analysis

The results were expressed as the mean ± standard deviation (SD), ranges, median, or percentages. Continuous variables were represented as the mean ± SD, unless otherwise stated. Categorical variables were represented as frequency analysis (*n* (%)). All statistical analyses were performed using the IBM Statistical Package for the Social Sciences (SPSS) Statistics for Windows, version 19 (IBM Corp., Armonk, N.Y., USA).

## 4. Ethics Considerations

This study was approved by the institutional review board of the Research Ethic Committee of Changhua Christian Hospital, in Taiwan (CCH IRB No. 210202).

## 5. Results

A total of 55 patients with Meckel's diverticulum diagnosed by BAE were enrolled in the current study. The clinical characteristics of all patients with Meckel's diverticulum are summarized in Table 1. The mean age of all patients with Meckel's diverticulum was 34.1 ± 17.4 years (range: 4 to 85 years). A total of 44 patients (80%, 44/55) were ≥20 years old at the time of diagnosis compared with 11 patients (20%, 11/55) who were <20 years old. There were 46 male patients (83.6%, 46/55) and 9 female patients (16.4%, 9/55) with a male: female ratio of 5.1 : 1. Most patients (78.2%, 43/55) had no major comorbid diseases on presentation while 12 patients did (21.8%, 12/55; 3 had hypertension and heart disease, 2 had thalassemia, 1 had chronic kidney disease, 1 had Crohn's disease, 1 had idiopathic thrombocytopenic purpura post-splenectomy, 1 had diabetes mellitus, 1 had liver cirrhosis, 1 had chronic hepatitis B, 1 had metastatic non-small-cell lung cancer, 1 had benign prostate hyperplasia, 1 had type B aortic dissection, 1 had old stroke, and 1 had a lung transplant for bronchiectasis). Overt GI bleeding (87.3%, 48/55) was the primary indicator for BAE in the majority of patients, followed by abdominal pain in 5 patients (9.1%, 5/55), suspected small bowel tumor in 1 patient (1.8%, 1/55), and Crohn's disease follow-up in 1 patient (1.8%, 1/55). Forty-four patients (80%, 44/55) had symptoms for ≤6 months before their diagnosis of Meckel's diverticulum by BAE, while 11 patients (20%, 11/55) had symptoms for >6 months.

**Figure 1:**
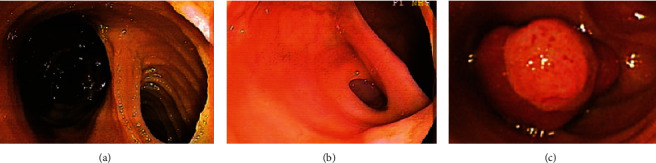
Endoscopy showing a large ostium of Meckel's diverticulum (a); endoscopy showing a small ostium of Meckel's diverticulum (b); endoscopy showing an inverted polypoid mass from a Meckel's diverticulum (c).

**Figure 2:**
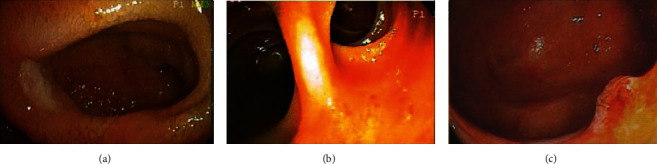
Endoscopy showing an ulcer in the margin of Meckel's diverticulum (a); endoscopy showing several erosions in the orifice of Meckel's diverticulum (b); endoscopy showing a protruding vessel in the margin of Meckel's diverticulum (c).

The diagnostic modalities and methods, endoscopic features, and anatomic findings for all patients with Meckel's diverticulum diagnosed by BAE are summarized in Table 2. We found that 29 patients (52.7%, 29/55) with Meckel's diverticulum were diagnosed using DBE while 26 patients (47.3%, 26/55) were diagnosed using SBE. Meckel's diverticulum was diagnosed via the retrograde approach in 54 patients (98.2%, 54/55) and was only diagnosed via the antegrade approach in 1 patient (1.8%, 1/55). In the analysis of endoscopic features, we found that 49 patients (89.1%, 49/55) with Meckel's diverticulum presented with a big ostium (Figure 1(a)), 4 patients (7.3%, 4/55) presented with a small ostium (Figure 1(b)), and 2 patients (3.6%, 2/55) presented with an inverted polypoid mass (Figure 1(C)). Following an analysis of mucosal changes in the Meckel's diverticulum, we identified 43 patients (78.2%, 43/55) who had mucosal ulcers (Figure 2(a)), erosions (Figure 2(b)), or visible vessels (Figure 2(c)) in the margin or inside of Meckel's diverticulum, indicating evidence of recent bleeding, while we found 12 patients (21.8%, 12/55) showed no mucosal ulcers, erosions, or visible vessels. Meckel's diverticula were located in the ileum of all patients within the study, and we found that the mean distance between the ileocecal valve and Meckel's diverticulum was 71.6 ± 33.6 cm (range, 25 cm to 200 cm). We also found that this distance was ≤60 cm in 32 patients (58.2%, 32/55) and >60 cm in 23 patients (41.8%, 23/55). The mean length of Meckel's diverticulum in the 42 patients who had it surgically resected was 5.2 ± 2.1 cm (range, 2 cm to 12 cm).

**Figure 3:**
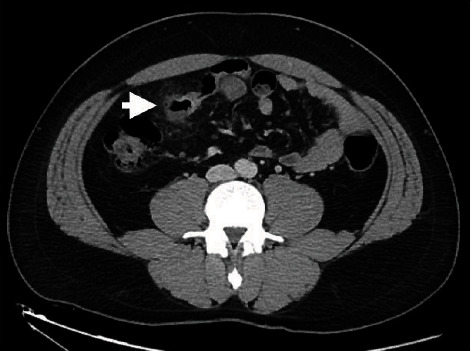
Abdominal computed tomography showing a blind-ending gas-filled structure with surrounding fat stranding and in continuity with small bowel from the antimesenteric border of the ileum (arrow).

**Figure 4:**
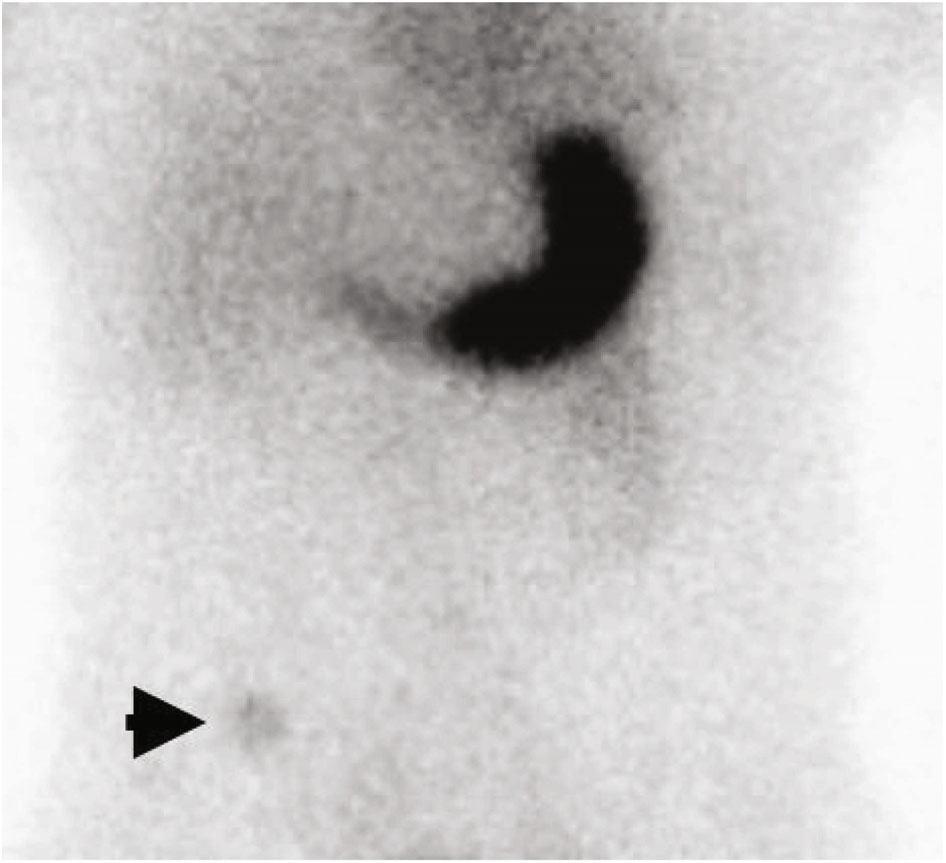
Technetium-99 m pertechnetate showing uptake (arrow) of ectopic gastric mucosa in the right lower quadrant of the abdomen, confirming the diagnosis of Meckel's diverticulum.

**Figure 5:**
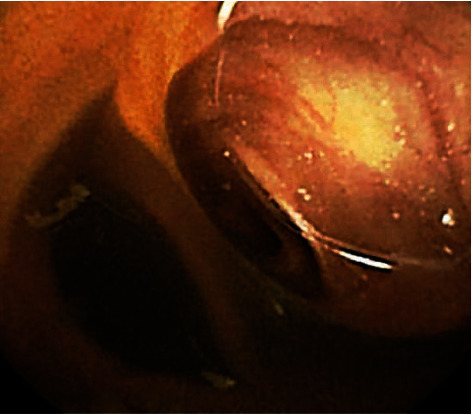
Capsule endoscopy showing two intestinal lumens in the distal ileum.

**Figure 6:**
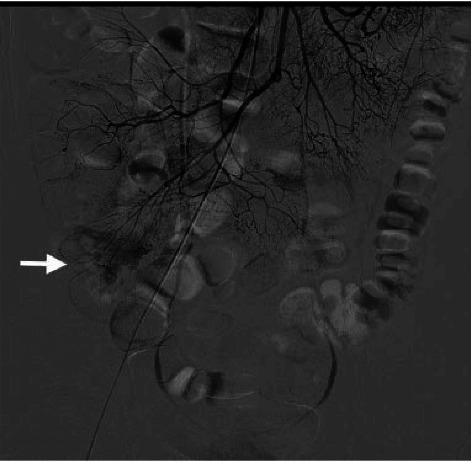
Digital angiography showing a contrast extravasation (arrow) from one of the branches of the superior mesenteric artery, confirming a bleeding Meckel's diverticulum.

The diagnostic yields of the different modalities, treatment methods, histopathological findings, and clinical outcomes of patients with Meckel's diverticulum diagnosed by BAE are summarized in Table 3. With regard to the detection rates of other diagnostic modalities, abdominal computed tomography (CT) was performed in 49 of the 55 patients (89.1%), but only 7 (14.3%, 7/49) had positive findings (Figure 3); technetium-99 m pertechnetate scintigraphy (so-called Meckel's scan) was performed in 29 of the 55 patients (52.7%), but only 10 (34.5%, 10/29) had positive findings (Figure 4); a small bowel series was performed in 16 of the 55 patients (29.1%), but only 2 (12.5%, 2/16) had positive findings; capsule endoscopy (CE) was performed in 11 of the 55 patients (20%), but only 5 (45.5%, 5/11) had positive findings (Figure 5); digital angiography was performed in 9 of the 55 patients (16.4%), but only 1 (11.1%, 1/9) had a positive finding (Figure 6).

Following an analysis of the treatment methods, our results showed that 42 patients (76.4%, 42/55) underwent surgical resection for Meckel's diverticulum following their endoscopic diagnosis. In the surgical treatment group, a laparoscopy was performed in 28 patients (66.7%, 28/42), a laparotomy was performed in 13 patients (30.9%, 13/42), and a laparoscopy which converted to laparotomy was performed in 1 patient (2.4%, 1/42). All 42 patients in the surgical treatment group underwent either wedge diverticulum resection (diverticulectomy) or partial ileal resection. Only 1 patient (2.4%, 1/42) had postoperative complication due to anastomotic ulcer bleeding diagnosed by DBE 1 month after segmental resection of small bowel. The patient experienced several episodes of recurrent bleeding, but his bleeding ultimately subsided after conservative treatment. There were 13 patients (23.6%, 13/55) who received conservative treatment because they refused surgery. In the conservative treatment group, patients with Meckel's diverticulum showed no recurrent bleeding with a mean follow-up period of 56.8 months (range 12-156 months) after endoscopic diagnosis excepting only 1 patient (7.7%, 1/13) had two events of recurrent GI bleeding during a follow-up period.

We analyzed the 43 patients who had histopathological findings from their endoscopic biopsies or surgical resection specimens. We found that heterotopic gastric tissue was identified in 19 patients (44.2%, 19/43), heterotopic gastric and pancreatic tissues were identified in 3 patients (7%, 3/43), heterotopic pancreatic tissue was identified in 2 patients (4.7%, 2/43), heterotopic colonic tissue was identified in 1 patient (2.3%, 1/43), and neuroendocrine grade 2 tumor was identified in 1 patient (2.3%, 1/43).

## 6. Discussion

Meckel's diverticulum is the most common congenital malformation of the GI tract. The prevalence of Meckel's diverticulum is usually equally distributed across both sexes, but there is a male predominance in symptomatic patients, with a male to female ratio ranging from 2 : 1 to 5 : 1 [11, 12]. In the current study, we found that the ratio of males to females was 5.1 : 1 for patients with Meckel's diverticulum who were diagnosed by BAE. Complications of Meckel's diverticulum have been reported to occur in 4-40% of patients and can include GI bleeding, intussusception, intestinal obstruction, diverticulitis, enteroliths, perforation, and, very rarely, vesico diverticular fistula and tumors [4]. Among these complications, GI bleeding occurs predominantly in children, while inflammation and obstructions tend to occur in adults [3–5, 7]. However, it is not uncommon for a diagnosis of Meckel's diverticulum to be missed in adults. When patients present with obscure GI bleeding accompanied by Meckel's diverticulum, it is often difficult to determine whether Meckel's diverticulum is the cause of the bleeding because Meckel's diverticula are usually asymptomatic. Therefore, additional information based on endoscopic observations and the features of the Meckel's diverticulum are required.

In the past, preoperative diagnosis of Meckel's diverticulum was a challenge for most endoscopists. Conventional diagnostic modalities for Meckel's diverticulum include a small bowel series, abdominal CT or magnetic resonance imaging (MRI), digital angiography, Meckel's scan, and even surgery. On a small bowel series, Meckel's diverticulum may manifest as a blind-ending pouch or a polypoid filling defect arising from the antimesenteric side of the ileum [13]. However, Meckel's diverticulum can be misdiagnosed via this method because of a small ostium, its filling with intestinal contents, or peristalsis with rapid emptying. Moreover, detection of ulcerations in a Meckel's diverticulum using a small bowel series is not usually possible. In the current study, we performed a small bowel series in 29.1% of patients; however, its diagnostic yield was only 12.5%. On abdominal CT or MRI, Meckel's diverticulum may be shown as a blind-ending fluid or gas-filled structure in continuity with the small intestine, but it is also difficult to distinguish from the normal small intestine in uncomplicated cases [14]. Our results showed that abdominal CT was the most commonly used diagnostic modality in patients with Meckel's diverticulum (89.1%); however, its diagnostic yield was only 14.3%. On digital angiography, a persistent vitellointestinal artery can be observed in most patients with Meckel's diverticulum who present with chronic GI bleeding [15]. This procedure can also be useful for applying embolization treatment for an overt bleeding vessel. In the current study, we performed a digital angiography in 16.4% of all patients, and it had a diagnostic yield of 11.1%. Meckel's scan is a useful modality for detecting the existence of Meckel's diverticulum because of its reactivity with the gastric mucosa. Although Meckel's scan showed a high sensitivity rate (85-90%) in pediatric patients, it had a low sensitivity rate (<60%) in adult patients [16]. Furthermore, this test has a false positive rate of 15% and a false negative rate of 25% due to obstruction, inflammatory bowel disease, angioma, ectopic kidney, and urinary tract uptake [17]. Therefore, positive results from Meckel's scan do not guarantee that Meckel's diverticulum is responsible for the bleeding. In the current study, we performed Meckel's scan in 52.7% of all patients; however, it showed a diagnostic yield of just 34.5%, which is in line with previous reports in adult patients.

It is usually difficult to identify a Meckel's diverticulum via conventional push-type enteroscopy or a colonoscopy because they cannot reach the ileum. However, Liu et al. first diagnosed a Meckel's diverticulum using a colonoscopy in a patient with obscure GI bleeding, in whom the distance between the ileocecal valve and Meckel's diverticulum was 40 cm due to intestinal resection [18]. Moreover, some clinicians have diagnosed patients with Meckel's diverticulum using a colonoscopy because the diverticula inverted into the colon and ileocecal valve [19, 20]. The diagnosis of small bowel diseases has evolved dramatically over the past two decades, particularly due to the introduction of newly developed diagnostic modalities, including CE and BAE. However, the diagnosis of Meckel's diverticulum by CE or BAE is usually presented in case reports and there is a lack of large studies in the English literature. There are two primary reasons for this. First, symptomatic Meckel's diverticulum is rare in adult patients. Second, CE and BAE are rarely performed in children with symptomatic Meckel's diverticulum because most pediatricians lack or have fewer experiences of CE and BAE. Meckel's diverticulum diagnosed by CE or BAE therefore occurs less frequently.

CE is a noninvasive technique used to examine the entire small bowel. However, the diagnostic yield of CE in Meckel's diverticulum was limited until now. Mylonaki et al. first reported a Meckel's diverticulum detected by CE and described it as a “black hole” or having a “blood-filled” appearance [21]. Furthermore, it is usually difficult to detect an ulcer in a Meckel's diverticulum via CE because of its rapid peristalsis. However, Montemaggi et al. have shown a circular ulcer in a Meckel's diverticulum detected by CE [22]. Although CE can detect a Meckel's diverticulum with or without ulcers, it lacks the capacity to sample tissues and there is a risk of capsule retention within the Meckel's diverticulum [23]. Despite the fact that only a few patients (20%) underwent CE in the current study, the diagnostic yield of CE was up to 45.5%, which was the highest diagnostic yield of all diagnostic modalities except for BAE. In contrast to CE, BAE can not only detect a Meckel's diverticulum but also has the capacity for tissue sampling and endoscopic treatment. Yamamoto et al. first reported a case of Meckel's diverticulum diagnosed by DBE [8]. Manner et al. reported 3 cases of Meckel's diverticulum diagnosed by DBE [24]. Later, Shinozaki et al. reported 5 patients with Meckel's diverticulum diagnosed by DBE [25]. More recently, Fukushima et al. reported 10 patients with Meckel's diverticulum diagnosed by DBE [26]. Zhu et al. just published a report of 10 children patients with Meckel's diverticular bleeding diagnosed by DBE in 2021 [27]. In contrast to these previous small case series reports, He et al. reported a large case series of 64 patients with Meckel's diverticulum diagnosed by DBE before surgery in China [28]. Their results showed that the overall diagnostic yield for DBE was significantly greater than that of CE (84.6% vs. 7.7%). However, previously, the type of BAE used for the diagnosis of Meckel's diverticulum was almost always DBE. Recently, Wei et al. reported 6 patients with Meckel's diverticulum diagnosed by SBE [29]. In the current study, we reported a case series of 55 patients with Meckel's diverticulum diagnosed by BAE, including DBE and SBE, in Taiwan. We found that 52.7% of patients with Meckel's diverticulum were diagnosed by DBE, while 47.3% were diagnosed by SBE, which showed a slight predominance of DBE in the type of BAE conducted.

With regard to endoscopic features, Meckel's diverticulum usually presents as a large ostium but it can also present as a small ostium or inverted polypoid lesions [25, 29–31]. Our results showed that 89.1% of patients with Meckel's diverticulum presented as a large ostium, and 7.3% of patients presented as a small ostium, while only 3.6% of patients presented as an inverted polypoid mass. Shinozaki et al. suggested that detection of ulcers could be reliable evidence of GI bleeding from a Meckel's diverticulum; however, the mechanism of ulcer formation remains unclear despite several hypotheses [25]. In the past, several authors speculated that gastric acid secreted from the heterotopic gastric mucosa could cause the ulcerations [3, 32]. Helicobacter pylori infection was also identified and considered a cause of ulcerations of Meckel's diverticulum with heterotopic gastric mucosa [33]. However, the above two theories are currently doubted by other authors in the literature [34, 35]. Manner et al. subsequently suggested that mechanical irritation in the area of the tissue bridge between the ileal lumen and Meckel's diverticulum could lead to ulceration [24].

If Meckel's diverticulum with ulcers is detected by BAE, total enteroscopy to evaluate the entire small intestine may not be necessary. Conversely, other bleeding sources should be investigated when no ulcers are identified in Meckel's diverticulum. Patients with Meckel's diverticulum who do not experience recurrent bleeding after surgery could support this theory. In a pediatric study reported by Rutherford et al., they found that 81% of patients with complaints of GI bleeding had ulcers in the resected Meckel's diverticula [36]. Our results showed that 78.2% of all patients had ulcerations, erosions, or visible vessels in the margin or inside of Meckel's diverticulum as determined by BAE.

Meckel's diverticulum may harbor heterotopic tissues within its mucosa, including gastric, duodenal, colonic, pancreatic, and hepatobiliary tissues [2]. Among these heterotopic tissues, heterotopic gastric tissue is the most common in symptomatic Meckel's diverticulum (45-80%) [36]. Yamaguchi et al. reported that gastric mucosa was only identified in 30% of patients with Meckel's diverticulum and 62% of symptomatic Meckel's diverticula [5]. Neoplasms arising in a Meckel's diverticulum are very rare, accounting for just 3% of symptomatic cases [13]. Neuroendocrine tumors are the most common, but other neoplasms including neuromas, lipomas, leiomyomas, leiomyosarcomas, angiomas, carcinosarcomas, and adenocarcinomas have also been previously reported [13, 37]. In the current study, heterotopic gastric mucosa was identified in 52.4% of all patients with surgically resected specimens or endoscopic biopsy specimens. Furthermore, we identified heterotopic pancreatic tissue in 5 patients (11.9%, 5/42), heterotopic colonic tissue in 1 patient (2.4%, 1/42), and a neuroendocrine tumor in 1 patient (2.4%, 1/42).

Conventionally, surgery has been the mainstay treatment for patients with complicated Meckel's diverticulum [38, 39]. Laparoscopy or laparoscopy-assisted treatment is a recognized safe and effective method for treating complicated Meckel's diverticulum compared with the alternative open approach [40, 41]. Our results showed that 76.4% of all patients with Meckel's diverticulum underwent surgical treatment, including laparoscopy (66.7%), laparotomy (30.9%), and laparoscopy converted to laparotomy (2.4%). Conservative treatment for Meckel's diverticulum haven reported in the English literature, especially for those patents, was discovered incidentally [42]. In our current study, 42 patients underwent either wedge diverticulum resection or partial ileal resection and only 1 patient (2.4%) had postoperative complication. However, 25.5% of patients underwent conservative treatment because they refused surgery. It should be noted that surgical treatment for asymptomatic and incidentally discovered Meckel's diverticulum remains controversial. McKay suggested that surgical resection of asymptomatic Meckel's diverticulum should be considered in patients < 50 years of age, whereas patients > 50 years of age would be less likely to benefit from this prophylactic resection [43]. Park et al. reported a study of 1,476 patients with Meckel's diverticulum diagnosed during surgery and suggested that surgical resection should only be performed when at least one of the following criteria was met: (1) patient age < 50 years; (2) male patient; (3) the length of diverticulum > 2 cm; and (4) the presence of histologically abnormal tissue in the diverticulum [44].

## 7. Conclusions

The results of the current study revealed that although complicated Meckel's diverticulum is a rare event, it should be considered in adult patients presenting with GI symptoms, especially younger patients with overt obscure GI bleeding. Abdominal CT scan has its diagnostic limitation to diagnose patients with Meckel's diverticulum despite the fact that it is the most commonly used modality before performing a BAE. On the contrary, BAE is a very useful modality for the detection of Meckel's diverticulum compared with other conventional diagnostic modalities; especially, retrograde BAE should be considered. Endoscopic detection of ulcers in a Meckel's diverticulum is important evidence of GI bleeding. Minimally invasive laparoscopic resection after an endoscopic diagnosis can be performed for the treatment of Meckel's diverticulum. The chief limitation of the current study was the small number of cases. We believe that further widespread usage of BAE will provide additional insights into the treatment of Meckel's diverticulum.

## Figures and Tables

**Table 1:** Clinical characteristics of patients with Meckel's diverticulum diagnosed by BAE (*n* = 55).

Patient characteristics	No. of patients (%)
Gender	
Male	46 (83.6)
Female	9 (16.4)
Age	
Mean age ± SD, years [range]	34.1 ± 17.4 [4-85]
≧20 years	44 (80)
<20 years	11 (20)
Symptoms	
Overt GI bleeding	48 (87.3)
Abdominal pain	5 (9.1)
Suspected small bowel tumor	1 (1.8)
Crohn's disease follow-up	1 (1.8)
Symptom onset	
Acute (≦6 months)	44 (80)
Chronic (>6 months)	11 (20)
Comorbidities	
Healthy	43 (78.2)
Comorbidity	12 (21.8)
HTN and heart disease	3
Thalassemia	2
Liver cirrhosis and CHB	2
Crohn's disease	1
CKD	1
ITP	1
BPH	1
Type B aortic dissection	1
Bronchiectasis postlung transplant	1
Metastatic non-small-cell lung cancer	1
Old stroke	1

BAE: balloon-assisted enteroscopy; BPH: benign prostate hyperplasia; CHB: chronic hepatitis B; CKD: chronic kidney disease; DM: diabetes mellitus; GI: gastrointestinal; HTN: hypertension; ITP: idiopathic thrombocytopenic purpura; SD: standard deviation.

**Table 2:** The diagnostic modalities and methods, endoscopic features, and anatomic appearances of patients with Meckel's diverticulum diagnosed by BAE (*n* = 55).

Patient characteristics	No. of patients (%)
Type of BAE	
Double-balloon enteroscopy	29 (52.7)
Single-balloon enteroscopy	26 (47.3)
Insertion direction of BAE	
Retrograde approach	54 (98.2)
Antegrade approach	1 (1.8)
Location of Meckel's diverticulum∗	
Antimesenteric side	41 (100)
Mesenteric side	0 (0)
Pattern of diverticular orifice	
Big ostium	49 (89.1)
Small ostium	4 (7.3)
Polypoid mass	2 (3.6)
Bleeding sign of Meckel's diverticulum	
Mucosal ulcerations or erosions	43 (78.2)
No	12 (21.8)
Distance between the ileocecal valve and Meckel's diverticulum^#^	
Mean distance ± SD, cm [range]	
≦60 cm	71.8 ± 33.2 [25-200]
>60 cm	32 (58.2)
Length of Meckel's diverticulum^∗^	23 (41.8)
Mean length ± SD, cm [range]	5.3 ± 2.1 [2-12]

BAE: balloon-assisted enteroscopy; SD: standard deviation. ^#^The measuring method was on a surgically resected specimen or estimated during BAE. ^∗^41 surgically resected patients.

**Table 3:** Diagnostic yield of different modalities, treatment methods, histopathological findings, and clinical outcomes of patients with Meckel's diverticulum diagnosed by BAE (*n* = 55).

Patient characteristics	No. of patients (%)
Diagnostic procedure used	
BAE	55 (100)
Abdominal CT	49 (89.1)
Meckel's scan	29 (52.7)
Small bowel series	16 (29.1)
Capsule endoscopy	11 (20)
Digital angiography	9 (16.4)
Yield of diagnostic procedure	
BAE	55/55 (100)
Capsule endoscopy	5/11 (45.5)
Meckel's scan	10/29 (35.7)
Abdominal CT	7/49 (14.6)
Small bowel series	2/16 (12.5)
Digital angiography	1/9 (11.1)
Treatment methods	
Surgical treatment	42 (76.4)
Conservative treatment	13 (23.6)
Heterotopic tissue^#^	
Gastric mucosa	19 (42.4)
Gastric and pancreatic tissues	3 (7)
Pancreatic tissue	2 (4.7)
Colonic mucosa	1 (2.3)
Neuroendocrine tumor	1 (2.3)
No	17 (39.5)

BAE: balloon-assisted enteroscopy; CT: computed tomography. ^#^43 patients with endoscopic biopsies or surgical resection specimens of Meckel's diverticulum.

## Data Availability

The datasets generated and/or analyzed during the current study are available from the corresponding author on reasonable request.
